# Surveillance To Track Progress Toward Polio Eradication — Worldwide, 2022–2023

**DOI:** 10.15585/mmwr.mm7313a1

**Published:** 2024-04-04

**Authors:** Nishant Kishore, Elizabeth Krow-Lucal, Ousmane M. Diop, Jaume Jorba, Tigran Avagnan, Varja Grabovac, Anfumbom K.W. Kfutwah, Ticha Johnson, Sudhir Joshi, Lucky Sangal, Salmaan Sharif, Ashraf Wahdan, Graham F. Tallis, Stephanie D. Kovacs

**Affiliations:** ^1^Global Immunization Division, Center for Global Health, CDC; ^2^World Health Organization, Geneva, Switzerland; ^3^Division of Viral Diseases, National Center for Immunization and Respiratory Diseases, CDC; ^4^World Health Organization Regional Office for the Western Pacific, Manila, Philippines; ^5^World Health Organization Regional Office for Africa, Brazzaville, Republic of the Congo; ^6^World Health Organization Regional Office for South-East Asia, New Delhi, India.; ^7^World Health Organization Regional Office for the Eastern Mediterranean, Amman, Jordan.

**Summary**

**What is already known about this topic?**

The primary means for detecting poliovirus is through surveillance for acute flaccid paralysis (AFP), supplemented by environmental surveillance of sewage samples.

**What is added by this report?**

During 2022–2023, among 28 priority countries experiencing or at high risk for poliovirus transmission, 20 (71.4%) met national AFP surveillance indicator targets, and the number of environmental surveillance sites in priority countries increased. However, substantial national and subnational AFP surveillance gaps persist.

**What are the implications for public health practice?**

Maintaining high-quality surveillance is critical to achieving the goal of global polio eradication. Monitoring surveillance indicators is important to identifying gaps and guiding surveillance strengthening activities, particularly in countries at high risk for poliovirus circulation.

## Abstract

The reliable and timely detection of poliovirus cases through surveillance for acute flaccid paralysis (AFP), supplemented by environmental surveillance of sewage samples, is a critical component of the polio eradication program. Since 1988, the number of polio cases caused by wild poliovirus (WPV) has declined by >99.9%, and eradication of WPV serotypes 2 and 3 has been certified; only serotype 1 (WPV1) continues to circulate, and transmission remains endemic in Afghanistan and Pakistan. This surveillance update evaluated indicators from AFP surveillance, environmental surveillance for polioviruses, and Global Polio Laboratory Network performance data provided by 28 priority countries for the program during 2022–2023. No WPV1 cases have been detected outside of Afghanistan and Pakistan since August 2022, when an importation into Malawi and Mozambique resulted in an outbreak during 2021–2022. During 2022–2023, among 28 priority countries, 20 (71.4%) met national AFP surveillance indicator targets, and the number of environmental surveillance sites increased. However, low national rates of reported AFP cases in priority countries in 2023 might have resulted from surveillance reporting lags; substantial national and subnational AFP surveillance gaps persist. Maintaining high-quality surveillance is critical to achieving the goal of global polio eradication. Monitoring surveillance indicators is important to identifying gaps and guiding surveillance-strengthening activities, particularly in countries at high risk for poliovirus circulation.

## Introduction

Since the Global Polio Eradication Initiative (GPEI) was established in 1988, the number of wild poliovirus (WPV) cases has declined by >99.9%, and WPV serotypes 2 and 3 have been declared eradicated (*1*). By the end of 2023, WPV type 1 (WPV1) transmission remained endemic only in Afghanistan and Pakistan (*2*,*3*). However, during 2021–2022, Malawi and Mozambique reported nine WPV1 cases caused by a virus genetically linked to cases from Pakistan (last paralysis onset date on August 10, 2022) (*4*,*5*). In areas with low polio vaccination coverage, prolonged circulation of vaccine-derived polioviruses (VDPV) can result in their reversion to neurovirulence. Infection with these circulating VDPVs (cVDPVs) can cause paralysis and polio outbreaks; cVDPV outbreaks have been detected in 42 countries (*6*).

Polioviruses are detected primarily through surveillance for acute flaccid paralysis (AFP), confirmed through stool specimen testing. Environmental surveillance (ES), the systematic sampling of sewage and testing for the presence of poliovirus, supplements AFP surveillance by detecting poliovirus circulation independent of confirmed paralytic polio cases. This report updates previous reports (*7*,*8*) to describe polio surveillance performance during 2022–2023 in 28 priority countries (i.e., those deemed to be at high risk for poliovirus transmission because of ongoing surveillance gaps and vulnerability to poliovirus circulation).*

## Methods

### Data Sources

Data analyzed in this study were obtained from 1) the World Health Organization (WHO) Polio Information System as of March 11, 2024, and 2) the Global Polio Laboratory Network (GPLN) as of January 31, 2024. These data are the property of the individual countries, and data access was provided through the GPEI Data Sharing Agreement.

### Acute Flaccid Paralysis and Environmental Surveillance

AFP surveillance quality was assessed for 28 priority countries both at the national level and at 511 first administrative subnational (i.e., state or province) level using two performance indicators: 1) the nonpolio AFP (NPAFP) rate^†^ (an NPAFP rate of two or more NPAFP cases per 100,000 persons aged <15 years indicates AFP surveillance is sufficiently sensitive to detect circulating poliovirus), and 2) stool adequacy (two stools collected within 14 days of paralysis onset, ≥24 hours apart, and received by a WHO-accredited laboratory via reverse cold chain and in good condition)^§^ with a target of ≥80% adequate stool specimens collected from AFP patients. ES site sensitivity to detect poliovirus is assessed by the annual enterovirus isolation rate, defined as the percentage of specimens with enterovirus detected, with a target of ≥50%.

### Global Polio Laboratory Network

The GPLN consists of 144 WHO-accredited laboratories in the six WHO regions, monitored through a standardized quality assurance program of annual onsite audits and proficiency testing (*9*). All 144 GPLN laboratories are responsible for isolating polioviruses; 134 conduct intratypic differentiation to identify WPV, VDPV, and Sabin (oral poliovirus vaccine) polioviruses; 28 laboratories conduct genomic sequencing.

## Analysis

R software (version 4.3.1; R Foundation) was used to conduct all analyses. All administrative boundaries, as well as the disputed borders, and the lakes within the disputed areas dataset were sourced from the WHO and GPEI administrative boundary project (https://polioboundaries-who.hub.arcgis.com/). This activity was reviewed by CDC, deemed not research, and was conducted consistent with applicable federal law and CDC policy.^¶^

## Results

### Acute Flaccid Paralysis

Surveillance indicators and detected cases were assessed in 28 priority countries during 2022–2023 (Table 1). Priority countries include 21 in the African region, five in the Eastern Mediterranean region, and one each in the South-East Asia and Western Pacific regions.

**TABLE 1:** National and subnational acute flaccid paralysis surveillance performance indicators and number of confirmed wild poliovirus and circulating vaccine-derived poliovirus cases, by country — 28 priority countries, World Health Organization African, Eastern Mediterranean, South-East Asia, and Western Pacific regions, 2022 and 2023*

Year/WHO region/ Country	No. of AFP cases (all ages)	Regional/ National NPAFP rate^†^	%				No. of confirmed cases	
			Subnational areas with NPAFP rate of two or more cases^§^	Regional/ National no. of AFP cases with adequate stool specimens^¶^	Subnational areas with ≥80% adequate stool specimens^§,¶^	Subnational areas meeting both indicators^§,¶,^**	WPV cases	cVDPV cases (type 1, type 2)^††^
**2022**								
**AFR (N = 21)**	27,786	7.1	N/A	90.6	N/A	N/A	8	690 (192, 498)
Angola	386	2.4	66.7	89.6	83.3	75.9	—^§§^	—
Botswana	32	3.5	45.8	78.1	33.3	24.5	—	—
Burkina Faso	1,260	12.4	100.0	93.0	100.0	100.0	—	—
Burundi	128	2.1	50.0	87.5	77.8	37.1	—	1 (0, 1)
Cameroon	852	7.1	100.0	81.9	60.0	62.1	—	3 (0, 3)
CAR	215	7.6	100.0	86.0	57.1	64.9	—	6 (0, 6)
Chad	1,254	14.3	100.0	82.1	52.2	52.4	—	44 (0, 44)
DRC	4,577	8.6	100.0	85.8	61.5	61.0	—	523 (150, 373)
Equatorial Guinea	388	6.7	100.0	88.9	100.0	100.0	—	—
Ethiopia	1,606	3.2	90.9	93.0	90.9	92.8	—	1 (0, 1)
Kenya	653	3.2	85.1	87.0	76.6	70.0	—	—
Madagascar	646	5.4	100.0	95.2	100.0	100.0	—	16 (16, 0)
Malawi	481	5.1	100.0	71.7	25.0	0.1	—	4 (4, 0)
Mali	562	5.3	100.0	87.2	90.9	99.5	—	2 (0, 2)
Mozambique	929	5.9	100.0	74.5	18.2	15.7	8	26 (22, 4)
Niger	991	7.6	100.0	87.8	75.0	74.3	—	16 (0, 16)
Nigeria	10,247	10.8	100.0	96.7	100.0	100.0	—	48 (0, 48)
South Sudan	557	11.4	100.0	93.9	100.0	100.0	—	—
Tanzania	1,283	4.5	93.5	98.1	100.0	98.6	—	—
Zambia	390	4.3	100.0	65.9	10.0	18.7	—	—
Zimbabwe	349	5.1	100.0	90.8	90.0	90.6	—	—
**EMR (N = 5)**	26,786	18.3	N/A	87.0	N/A	N/A	22	166 (0, 166)
Afghanistan	5,370	30.2	100.0	94.4	100.0	100.0	2	—
Pakistan	19,033	22.0	85.7	84.9	100.0	100.0	20	—
Somalia	356	4.2	90.5	97.2	95.2	96.7	—	5 (0, 5)
Sudan	650	3.4	100.0	97.1	94.4	98.1	—	1 (0, 1)
Yemen	1,377	9.1	100.0	81.0	60.9	59.7	—	160 (0, 160)
**SEAR (N = 1)**	2,412	3.5	N/A	73.7	N/A	N/A	—	1 (0, 1)
Indonesia	2,412	3.5	73.5	73.7	26.5	20.1	—	1 (0, 1)
**WPR (N = 1)**	63	1.8	N/A	65.1	N/A	N/A	—	—
Papua New Guinea	63	1.8	22.7	65.1	40.9	7.5	—	—
**2023**								
**AFR (N = 21)**	31,325	7.8	N/A	91.4	N/A	N/A	—	492 (133, 359)
Angola	482	2.7	77.8	84.6	72.2	77.9	—	—
Botswana	42	3.7	45.8	73.8	37.5	29.3	—	—
Burkina Faso	1,126	10.8	100.0	94.3	100.0	100.0	—	2 (0, 2)
Burundi	174	2.7	72.2	82.2	61.1	35.4	—	1 (0, 1)
Cameroon	855	7.0	100.0	87.6	80.0	87.9	—	—
CAR	209	6.8	100.0	80.9	57.1	63.3	—	15 (0, 15)
Chad	1,497	16.6	95.7	87.4	78.3	84.6	—	55 (0, 55)
DRC	4,674	9.1	100.0	83.3	69.2	69.1	—	223 (105, 118)
Equatorial Guinea	581	8.8	100.0	85.4	87.5	91.2	—	47 (0, 47)
Ethiopia	1,449	2.8	90.9	94.5	90.9	99.2	—	—
Kenya	693	3.2	83.0	88.0	70.2	64.3	—	8 (0, 8)
Madagascar	1,424	11.0	100.0	90.6	100.0	100.0	—	24 (24, 0)
Malawi	554	5.9	100.0	90.1	100.0	100.0	—	—
Mali	991	8.8	100.0	91.9	90.9	99.1	—	16 (0, 16)
Mozambique	676	4.2	100.0	81.5	72.7	77.7	—	5 (4, 1)
Niger	752	5.6	100.0	76.9	50.0	23.9	—	2 (0, 2)
Nigeria	12,020	12.4	100.0	97.3	100.0	100.0	—	87 (0, 87)
South Sudan	554	11.1	100.0	95.7	100.0	100.0	—	2 (0, 2)
Tanzania	1,551	5.2	100.0	97.0	100.0	100.0	—	3 (0, 3)
Zambia	682	6.6	100.0	79.2	70.0	71.0	—	1 (0, 1)
Zimbabwe	339	4.7	100.0	87.9	80.0	82.0	—	1 (0, 1)
**EMR (N = 5)**	27,794	18.6	N/A	86.1	N/A	N/A	11	15 (0, 15)
Afghanistan	5,852	32.3	100.0	94.0	100.0	100.0	5	—
Pakistan	19,714	22.3	85.7	83.9	85.7	98.7	6	—
Somalia	424	4.9	100.0	93.4	95.0	98.4	—	8 (0, 8)
Sudan	473	2.0	77.8	75.3	44.4	10.7	—	—
Yemen	1,331	9.4	100.0	84.2	87.0	83.1	—	7 (0, 7)
**SEAR (N = 1)**	4,362	5.8	N/A	73.3	N/A	N/A	—	6 (0, 6)
Indonesia	4,362	5.8	79.4	73.3	20.6	20.3	—	6 (0, 6)
**WPR (N = 1)**	61	1.3	N/A	54.1	N/A	N/A	—	—
Papua New Guinea	61	1.3	18.2	54.1	22.7	4.4	—	—

**Abbreviations:** AFP = acute flaccid paralysis; AFR = African Region; CAR = Central African Republic; cVDPV = circulating vaccine-derived poliovirus; DRC = Democratic Republic of the Congo; EMR = Eastern Mediterranean Region; N/A = not applicable; NPAFP = nonpolio acute flaccid paralysis; SEAR = South-East Asia Region; WHO = World Health Organization; WPR = Western Pacific Region; WPV = wild poliovirus.

* Data as of March 11, 2024.

^†^ Per 100,000 persons aged <15 years per year.

^§^ For all subnational areas regardless of population size.

^¶^ Surveillance targets are two or more NPAFP cases per 100,000 persons aged <15 years per year and ≥80% of persons with AFP having two stool specimens collected within 14 days of paralysis onset and ≥24 hours apart and received in good condition (i.e., without leakage or desiccation) by a WHO-accredited laboratory via reverse cold chain (storing and transporting samples at recommended temperatures from the point of collection to the laboratory).

** Percentage of the country’s population aged <15 years living in subnational areas that met both surveillance indicators (NPAFP rates of two or more per 100,000 persons aged <15 years per year and ≥80% of AFP cases with adequate specimens).

^††^ https://polioeradication.org/wp-content/uploads/2016/09/Reporting-and-Classification-of-VDPVs_Aug2016_EN.pdf

^§§^ Dashes indicate that no confirmed cases were found.

**African Region.** Among the 21 priority countries in the WHO African Region (AFR), 18 (85.7%) met both surveillance indicator targets nationally in 2023, compared with 17 (81%) in 2022. In 2023, all countries met the NPAFP rate target of two or more NPAFP cases per 100,000 persons aged <15 years.

In 2022 and 2023, 70.8% of 356 and 75.8% of 355 subnational regions, respectively, met both targets. Eleven countries reported that ≥80% of subnational regions met both indicators in 2023 (Figure) compared with nine countries in 2022.

**FIGURE:**
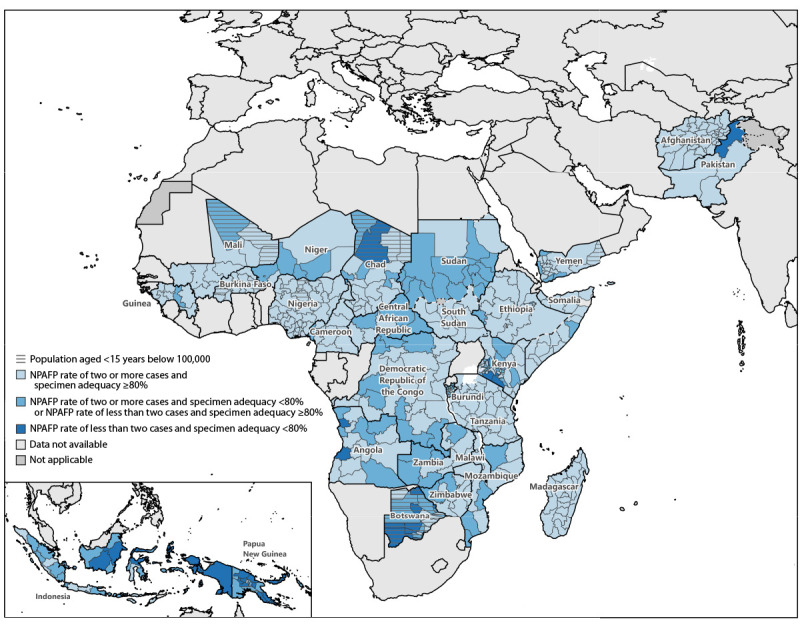
Combined performance indicators for the quality of acute flaccid paralysis surveillance*^,^^†^ in subnational areas of 28 priority countries^§^^,^^¶^ — World Health Organization African, Eastern Mediterranean, South-East Asia, and Western Pacific regions, 2023** **Abbreviations:** AFP = acute flaccid paralysis; NPAFP = nonpolio acute flaccid paralysis; WHO = World Health Organization. * The number of NPAFP cases per 100,000 children aged <15 years per year. NPAFP cases are cases of AFP determined not to be polio upon further case investigation and stool testing. The threshold of two or more NPAFP cases indicating that AFP surveillance is sufficiently sensitive to detect circulating polio is based on a background rate of AFP due to etiologies other than polioviruses. ^†^ Surveillance targets are two or more NPAFP cases per 100,000 persons aged <15 years per year and ≥80% of persons with AFP having two stool specimens collected within 14 days of paralysis onset, ≥24 hours apart, and received in good condition (i.e., without leakage or desiccation) by a WHO-accredited laboratory via reverse cold chain (storing and transporting samples at recommended temperatures from the point of collection to the laboratory). ^§^ The 2023 priority countries include the following: *African Region* (21): Angola, Botswana, Burkina Faso, Burundi, Cameroon, Central African Republic, Chad, Democratic Republic of the Congo, Ethiopia, Equatorial Guinea, Kenya, Madagascar, Malawi, Mali, Mozambique, Niger, Nigeria, South Sudan, Tanzania, Zambia, and Zimbabwe; *Eastern Mediterranean Region* (five): Afghanistan, Pakistan, Somalia, Sudan, and Yemen; *South-East Asia Region* (one): Indonesia; *Western Pacific Region* (one): Papua New Guinea. ^¶^ NPAFP rate is difficult to interpret when the population aged <15 years is below 100,000. ** Dotted and dashed lines on maps represent approximate border lines for which there might not yet be full agreement.

Eight WPV1 cases were detected in 2022 linked to one reported imported WPV1 case with onset in 2021; no WPV1 cases were reported in 2023. The number of VDPV cases decreased from 690 (192 cVDPV type 1 [cVDPV1] and 498 cVDPV type 2 [cVDPV2]) in 2022 to 471 (133 cVDPV1 and 338 cVDPV2) in 2023.

**Eastern Mediterranean Region.** Among the five priority countries in the WHO Eastern Mediterranean Region (EMR), all met both national surveillance indicator targets in 2022, and four met targets in 2023. Whereas 87.4% of subnational areas across the entire region met both indicator targets in 2022, the percentage declined to 80.4% in 2023. As of the reporting date, 11 WPV1 and 15 cVDPV2 cases were reported in 2023 compared with 22 and 166, respectively, in 2022.

**South-East Asia Region.** The WHO South-East Asia Region (SEAR) includes one priority country (Indonesia). At the national level, the NPAFP rate increased from 3.5 to 5.8 cases per 100,000; the percentage of stool samples that were adequate did not meet the indicator in either 2022 or 2023. Indonesia reported six cVDPV2 cases in 2023 compared with one case in 2022.

**Western Pacific Region.** The WHO Western Pacific Region includes one priority country (Papua New Guinea); neither national surveillance indicator target was met during this assessment period. No poliovirus was detected in Papua New Guinea during 2022–2023.

### Environmental Surveillance

In 2023, 27 (96.4%) of the 28 priority countries** had at least one ES site reporting. In priority countries in AFR, the number of ES sites decreased 2%, from 386 in 2022 to 378 in 2023; however, the proportion of sites meeting the enterovirus sensitivity target increased 41%, from 41.7% to 58.8%. In 2022 and 2023, ≥80% of sites in 18 and 19 countries, respectively, met the ≥50% enterovirus isolation rate target.

The number of ES sites in EMR increased 134%, from 244 in 2022 to 571 in 2023; this increase was driven by Pakistan, which added 308 new ES sites in 2023. However, only 133 (26.7%) of all ES sites in Pakistan reported five or more collections in 2023. In Somalia and Sudan, the proportion of sites meeting the sensitivity indicator declined from 100% to 35.3% and from 85.7% to 60%, respectively.

In Indonesia, the only priority country evaluated in SEAR, the number of ES sites decreased from 16 in 2022 to 12 in 2023. However, the proportion of sites that met the sensitivity indicator increased from 25% in 2022 to 45.5% in 2023.

### Global Polio Laboratory Network

In 2023, the GPLN tested 233,437 stool specimens collected from patients with AFP (Table 2). All WHO regions except the Region of the Americas met the timeliness target for viral isolation (results reported for ≥80% of specimens ≤14 days after receipt of specimen). All regions met the timeliness indicator for reporting (results reported for ≥80% of specimens within 7 days of receipt of isolates in the laboratory).

**TABLE 2:** Number of poliovirus isolates from stool specimens of persons with acute flaccid paralysis and timing of results, by World Health Organization region — worldwide, 2022 and 2023*^,^**^†^**

WHO region/ Year	No. of specimens	No. of poliovirus isolates			% of on-time^††^ poliovirus isolation test results	% ITD results	
		Wild^§^	Sabin^¶^	cVDPV**		Within 7 days of receipt at laboratory^§§^	Within 60 days of paralysis onset^¶¶^
**Africa**							
2022	53,961	8	3,065	453	86	85	83
2023	72,543	0	397	538	88	92	94
**Americas**							
2022	1,858	0	7	2	74	100	67
2023	1,826	0	2	0	72	100	100
**Eastern Mediterranean**							
2022	57,364	32	1,331	277	75	88	82
2023	76,322	19	2,033	9	92	100	79
**European**							
2022	2,980	0	22	2	79	91	91
2023	2,910	0	33	4	83	97	*92*
**South-East Asia**							
2022	67,118	0	1,067	2	96	98	93
2023	67,100	0	783	10	94	95	96
**Western Pacific**							
2022	10,664	0	32	0	98	100	100
2023	12,736	0	140	0	97	99	90
**Total^††^**							
2022*	**193,945**	**40**	**5,524**	**736**	**87**	**88**	**85**
2023**	**233,437**	**19**	**3,388**	**561**	**91**	**97**	**86**

**Abbreviations**: AFP = acute flaccid paralysis; cVDPV = circulating vaccine-derived poliovirus; ITD = intratypic differentiation; VDPV = vaccine-derived poliovirus; VP1 = poliovirus capsid viral protein 1; WHO = World Health Organization; WPV = wild poliovirus.

* Data for 2023 received from WHO regions during January 15–31, 2024.

^†^ Data for 2022 current as of January 31, 2023.

^§^ Number of AFP cases with WPV isolates.

^¶^ Either 1) concordant Sabin-like results in ITD test and VDPV screening, or 2) ≤1% VP1 nucleotide sequence difference compared with Sabin vaccine virus (≤0.6% for type 2).

** For poliovirus types 1 and 3, 10 or more VP1 nucleotide differences from the respective poliovirus; for poliovirus type 2, six or more VP1 nucleotide differences from Sabin type 2 poliovirus.

^††^ Results reported within 14 days of receipt of specimen.

^§§^ Results of ITD reported within 7 days of receipt of isolate.

^¶¶^ For percentage of poliovirus isolation results on time, percentage of ITD results within 7 days of receipt at laboratory and within 60 days of paralysis onset. Total represents weighted mean percentage of regional performance.

In genetic sequencing performed during 2022–2023, the South Asia genotype was the only circulating WPV1 isolated from 42 persons with AFP (30 in 2022 and 12 in 2023) in the two countries with endemic WPV1 transmission (Afghanistan and Pakistan) (*2*,*3*) and one person with AFP from Mozambique (*5*). In Pakistan, all 2022–2023 isolates were related to the YB3A genetic cluster (i.e., groups of polioviruses sharing ≥95% sequence identity in the region coding the viral capsid protein VP1) except three isolates in 2023, which were related to genetic cluster YB3C. In Afghanistan, all isolates were related to the YB3A genetic cluster. In Mozambique, eight WPV1 polio cases in 2022 were linked to the YC2 genetic cluster; no new WPV1 cases were detected in 2023. During the reporting period, cluster YB3A was detected in ES samples from Afghanistan and clusters YB3A and YB3C in ES samples from Pakistan; five ES detections (four in Pakistan and one in Afghanistan) were orphan viruses (i.e., isolates with ≥1.5% nucleotide divergence of the VP1-coding region from known isolates), indicating that virus circulation was prolonged.

In the 28 priority countries during 2022–2023, viruses from 37 cVDPV emergence groups (those not linked to any other outbreak, including seven cVDPV1 and 30 cVDPV2 emergence groups) were isolated from 1,320 AFP patients and 607 ES samples. The number of cVDPV1 emergence groups decreased from seven in 2022 to four in 2023. The number of cVDPV2 emergence groups increased from 18 in 2022 to 22 emergence groups in 2023.

## Discussion

Among 28 polio priority countries assessed during the 2022–2023 surveillance evaluation period, 20 (71.4%) met national AFP surveillance targets, and the total number of ES sites increased. Although the overall number of ES sites increased in EMR, Somalia and Sudan reported large decreases in the proportion of sites meeting the 50% enterovirus detection surveillance sensitivity indicator. The national NPAFP rate decreased in 14 of the 28 priority countries in 2023; Papua New Guinea did not meet the NPAFP target. Similarly, the percentages of adequate stool samples from persons with AFP declined in 15 (53.5%) priority countries; Botswana, Indonesia, Niger, Papua New Guinea, and Sudan did not reach target indicators.

The detection of imported WPV1 in Malawi and Mozambique in 2021–2022 highlights the importance of outbreak response in strengthening surveillance systems (*4*,*5*). Response to the 2021 WPV1 importation included Malawi, Mozambique, Tanzania, Zambia, and Zimbabwe. All response countries met the NPAFP rate target in 2023. Zambia reported a stool adequacy rate just below the target; however, the proportion of AFP cases with adequate specimens increased from 65.9% in 2022 to 79.2% in 2023. All subnational areas in Mozambique met the NPAFP target in 2022 and 2023, and the proportion of subnational regions meeting the stool adequacy target improved from 18.2% to 72.7% in 2023.

### Limitations

The findings in this report are subject to at least three limitations. First, metrics collected for this analysis can take weeks or months to be uploaded to the surveillance system and be available for analysis. Thus, whereas decreases in overall case numbers are encouraging, these trends might be affected by incomplete data resulting from surveillance lags. Second, performance measures reported at regional and national levels can obscure variation at lower administrative levels. Large populations residing in hard-to-reach areas might not be accessed by the surveillance system, which could affect the performance indicators and their interpretation. Finally, meeting performance indicators does not by itself ensure strong surveillance unless field activities are well supervised, and staff members are well trained.

### Implications for Public Health Practice

High-quality surveillance is crucial for the timely detection of circulating polioviruses and the rapid activation of outbreak response vaccination activities to stop transmission. Countries must monitor surveillance indicators to identify gaps and enhance the sensitivity and timeliness of surveillance activities through supportive supervision and training to guide progress toward the goal of global polio eradication.

## Footnotes

* Priority countries were included if they were deemed at high risk for poliovirus transmission in a country risk assessment exercise because of ongoing gaps in surveillance and vulnerability to poliovirus circulation, as described in the WHO Global Polio Surveillance Action Plan, 2022–2024 (https://polioeradication.org/wp-content/uploads/2022/05/GPSAP-2022-2024-EN.pdf). The priority countries are updated every year. The 2023 priority countries include the following: *African Region* (21): Angola, Botswana, Burkina Faso, Burundi, Cameroon, Central African Republic, Chad, Democratic Republic of the Congo, Equatorial Guinea, Ethiopia, Kenya, Madagascar, Malawi, Mali, Mozambique, Niger, Nigeria, South Sudan, Tanzania, Zambia, and Zimbabwe; *Eastern Mediterranean Region* (five): Afghanistan, Pakistan, Somalia, Sudan, and Yemen; *South-East Asia Region* (one): Indonesia; *Western Pacific Region* (one): Papua New Guinea.

† The number of NPAFP cases per 100,000 children aged <15 years per year. NPAFP cases are cases of AFP determined not to be polio upon further case investigation and stool testing. The threshold of two or more NPAFP cases indicating that AFP surveillance is sufficiently sensitive to detect circulating polio is based on a background rate of AFP due to etiologies other than polioviruses. The NPAFP rate is difficult to interpret when the population aged <15 years is below 100,000.

§ Two stool specimens that are collected from patients with AFP within 14 days of paralysis onset, ≥24 hours apart, and received in good condition (i.e., without leakage or desiccation) by a WHO-accredited laboratory via reverse cold chain (a transportation and storage method designed to keep samples at recommended temperatures from collection through arrival at the laboratory).

¶ 45 C.F.R. part 46.102(l)(2), 21 C.F.R. part 56; 42 U.S.C. Sect. 241(d); 5 U.S.C. Sect. 552a; 44 U.S.C. Sect. 3501 et seq.

** No ES sites were reported from Papua New Guinea during 2022–2023.

## References

[1] Lee SE, Greene SA, Burns CC, Tallis G, Wassilak SGF, Bolu O. Progress toward poliomyelitis eradication—worldwide, January 2021–March 2023. MMWR Morb Mortal Wkly Rep 2023;72:517–22. 10.15585/mmwr.mm7219a3 37167156 PMC10208367

[2] Bjork A, Akbar IE, Chaudhury S, Progress toward poliomyelitis eradication—Afghanistan, January 2022–June 2023. MMWR Morb Mortal Wkly Rep 2023;72:1020–6. 10.15585/mmwr.mm7238a1 37733636 PMC10519716

[3] Mbaeyi C, Baig S, Safdar RM, Progress toward poliomyelitis eradication—Pakistan, January 2022–June 2023. MMWR Morb Mortal Wkly Rep 2023;72:880–5. 10.15585/mmwr.mm7233a1 37590173 PMC10441828

[4] Global Polio Eradication Initiative. Malawi. Geneva, Switzerland: Global Polio Eradication Initiative; 2023. https://polioeradication.org/where-we-work/malawi/

[5] Global Polio Eradication Initiative. Mozambique. Geneva, Switzerland: Global Polio Eradication Initiative; 2023. https://polioeradication.org/where-we-work/Mozambique/

[6] Bigouette JP, Henderson E, Traoré MA, Update on vaccine-derived poliovirus outbreaks—worldwide, January 2021–December 2022. MMWR Morb Mortal Wkly Rep 2023;72:366–71. 10.15585/mmwr.mm7214a3 37022974 PMC10078846

[7] Wilkinson AL, Diop OM, Jorba J, Gardner T, Snider CJ, Ahmed J. Surveillance to track progress toward polio eradication—worldwide, 2020–2021. MMWR Morb Mortal Wkly Rep 2022;71:538–44. 10.15585/mmwr.mm7115a2 35421079 PMC9020859

[8] Stehling-Ariza T, Wilkinson AL, Diop OM, Surveillance to track progress toward poliomyelitis eradication—worldwide, 2021–2022. MMWR Morb Mortal Wkly Rep 2023;72:613–20. 10.15585/mmwr.mm7223a1 37289657 PMC10328463

[9] Diop OM, Kew OM, de Gourville EM, Pallansch MA. The Global Polio Laboratory Network as a platform for the viral vaccine-preventable and emerging diseases laboratory networks. J Infect Dis 2017;216(Suppl_1):S299–307. 10.1093/infdis/jix092 28838192 PMC5853949

